# Supplementary material for: New mammalian and avian records from the late Eocene La Meseta and Submeseta formations of Seymour Island, Antarctica
Source: PeerJ. 2020 Jan 9;8:e8268. doi: 10.7717/peerj.8268 (PMC6955110; doi:10.7717/peerj.8268)
Supplement: Figure S1 [file peerj-08-8268-s001.pdf]

Generated with Agisoft PhotoScan
